# Introduction and Systematic Review of the Good Nursing Care Scale

**DOI:** 10.1111/jocn.17486

**Published:** 2024-10-11

**Authors:** Tuula Mattila, Minna Stolt, Jouko Katajisto, Helena Leino‐Kilpi

**Affiliations:** ^1^ Department of Nursing Science, Faculty of Medicine University of Turku Turku Finland; ^2^ Turku University Hospital Turku Finland; ^3^ Satakunta Wellbeing Services County Pori Finland; ^4^ Department of Mathematics and Statistics University of Turku Turku Finland

**Keywords:** health care quality, instrument development, nursing care, patient‐centred care, quality of care

## Abstract

**Aim(s):**

To provide an introduction to the Good Nursing Care Scale (GNCS) and systematically review the application of the scale in health research.

**Design:**

Systematic review.

**Methods:**

Empirical studies published in English or Finnish in peer‐reviewed journals or as a summary of a PhD thesis where the scale was used for data collection amongst patients were included. Analysis was made by using descriptive statistics, narrative analysis, and evaluation of psychometric properties.

**Data Sources:**

PubMed, CINAHL, Cochrane, and Scopus in October 2023.

**Results:**

A total of 26 full‐text studies and summaries of PhD theses were included in the review. The GNCS has been developed systematically, and the theoretical structure has remained stable. The studies indicate a high level of patient‐centered quality of nursing care. Validity and reliability evaluation and reporting were systematic in the studies and mainly indicate sufficient level. Variations between countries are not large, supporting the international use of the GNCS.

**Conclusions:**

Patient‐centered quality of nursing care is predominantly at high levels. However, systematic evaluation is needed to provide longitudinal data. For that purpose, the GNCS is one potential instrument.

**Implications for the Profession and Patient Care:**

Support for the use of existing, tested instruments is encouraged to provide critical ideas for the future needs of nurse practitioners, managers, teachers and researchers.

**Impact:**

This paper impacts researchers interested in systematic evaluation of the patient‐centered quality of nursing care and for practitioners taking care of patients. For researchers, it introduces a relevant instrument, the GNCS, for analysing the quality or for comparing the quality with other instruments. For practitioners, it produces evidence of the usability of the GNCS.

**Reporting Method:**

PRISMA guided the systematic review, and the COSMIN guideline was used for quality appraisal of included studies.

**Patient or Public contribution:**

No Patient or Public contribution.

**Summary**

- What does this paper contribute to the wider global clinical community?
  ○ In this paper, evaluation of the quality of nursing care from the perspective of health service users, i.e., patients, is seen as fundamental for the evaluation of the health service system. For evaluation, we need valid patient‐centered instruments.
  ○ Instrumentation is the context of the study, and we report the development and psychometric quality of the GNCS, dating back to the 1990s and translated into several languages.
  ○ Our ultimate goal is to support the use of existing, tested instruments for gathering long‐term fundamental knowledge about the patient‐centered quality of nursing care as well as to provide critical ideas for the future needs of nurse practitioners, managers, teachers, and researchers. The GNCS is one valid option for this purpose.

## 1 Introduction

Evaluation of the quality of nursing care from the perspective of health service users, i.e., patients, is fundamental for the evaluation of the health service system as a whole. Health service is presumed to meet the needs and expectations of the population, and evaluative information from patients can optimise the practices. This basic assumption is generally described as patient‐centeredness in the evaluation of quality (OECD 2022; European Union 2021; WHO et al. 2018; Hanefeld, Powell‐Jackson, and Balabanova 2017), patients' perceptions being a central quality indicator (Wong, Mavondo, and Fisher 2020; WHO et al. 2018; Edvardsson, Watt, and Pearce 2017; Hanefeld, Powell‐Jackson, and Balabanova 2017).

Due to the diversity of the concept of quality, patients' evaluations and assessments of the quality of nursing care have focused on many different aspects. Quality can be seen as experiential, emphasising the nature of the individual experiences of patients (OECD 2022), well‐illustrated in the evaluation of satisfaction‐experience as an indicator of the quality of care (McCay, Lyles, and Larkey 2018; Sanchez‐Balcells et al. 2018; Leino‐Kilpi and Vuorenheimo 1992). This experience can have two roles in the evaluation: it can be seen as an outcome of quality or as a prerequisite for quality (Beattie et al. 2015). In addition to the experiential element, patient‐centered quality can be evaluated from the perspective of patient outcomes. From this perspective, quality of care is high if it supports the health empowerment of patients and the population (e.g., Jones and Meleis 1993). For its part, empowerment has several characteristics, the core being the power and ability to control one's own health and decide and act for it (e.g., Pekonen et al. 2019; EMPATHiE Consortium 2014; International Alliance of Patients' Organizations 2012).

Different definitions of quality require different patient‐centered evaluative scales to be used. Based on the scales, the quality of nursing care has usually been described both on a general, summative level and on the level of subcategories. Generally, patients have evaluated the quality to be at least at a moderate level (Al‐Awamreh and Suliman 2019; Karaca and Durna 2019; Naef, Ernst, and Petry 2019; Hatami et al. 2018; Sandsdalen et al. 2017; Charalambous et al. 2016; Desborough et al. 2015; Forsberg et al. 2015; Andersson and Lindgren 2013; Zuidgeest et al. 2012; Törnvall and Wilhelmsson 2010; Fröjd et al. 2011). On the contrary, low levels (Alsyouf et al. 2018) or neither satisfying nor dissatisfying levels of quality (Gishu, Weldetsadik, and Tekleab 2019) have also been identified. For example, in a large hospital‐based study (*n* = 182 hospitals, Aiken et al. 2017) in an average hospital, half of the patients gave low ratings on the quality of care. Furthermore, there seem to be areas that are more often given low ratings, i.e., areas related to patient information and education (Gishu, Weldetsadik, and Tekleab 2019; Karaca and Durna 2019; Forsberg et al. 2015; Fröjd et al. 2011). Improvement of the quality in patients' participation in their own care (Alsyouf et al. 2018; Forsberg et al. 2015; Fröjd et al. 2011; Törnvall and Wilhelmsson 2010) has also been reported. Moreover, physical care (Gishu, Weldetsadik, and Tekleab 2019), coordination of care (Naef, Ernst, and Petry 2019), continuity of care (Törnvall and Wilhelmsson 2010), or aspects related to the quality of the organisation (Al‐Awamreh and Suliman 2019; Andersson and Lindgren 2013) have been identified as needing development to promote the quality of nursing care. Thus, we have knowledge about patients' evaluations of the quality of nursing care. However, in order to obtain more systematic knowledge, it is important to have instruments that reflect the fundamentals of nursing care, have continuity, are used in different contexts, and employ rigorous research designs. Psychometrics generally incorporate the concepts of validity and reliability (DeVellis and Thorpe 2021; Mokkink et al. 2020). However, little is known about the psychometrics of the current patient‐centered evaluative scales.

The measurement scale of the Good Nursing Care Scale (GNCS) was developed for the assessment of the level of the quality of nursing care as seen by patients. The GNCS has two parallel parts, one for patients and the other for nurses. In this paper, we focus on the patient version of the GNCS. The aim is to provide an introduction to the GNCS and systematically review the application of the scale, created and used for the evaluation of the patient‐centered quality of nursing care since the 1990s.

## 2 Aim

The aim was to provide an introduction to the GNCS and systematically review the application of the scale, created and used for the evaluation of the patient‐centered quality of nursing care since the 1990s. To reach this aim, the following research questions were applied:

1. What is the level of the quality of nursing care as perceived by patients?
2. What variables, if any, relate to the level of quality of nursing care?
3. What are the psychometric properties of the GNCS?

## 3 Methods

For introducing the GNCS, the phases (1–7) of development and use of the scale from early 1990s to recent times were described, illustrating process of establishment of valid international instruments (e.g., Nunnally and Bernstein 1994; Leino‐Kilpi and Vuorenheimo 1993; DeVellis and Thorpe 2021; Goldman 2016). The aim, structure, content, and response scales are included into the introductory part.

For the review, a systematic review design was applied. A protocol for the systematic review was developed a priori and agreed to by the research team; however, it has not previously been published or registered. The PRISMA 2020 statement: an updated guideline for reporting systematic reviews guided the systematic review at all phases (Page et al. 2021, Appendices S1 and S2).

### 3.1 Search Strategy and Inclusion Criteria

To identify studies using the GNCS, database searches were conducted in October 2023 in Medline (PubMed), CINAHL (Ebsco), Cochrane library, and Scopus (Elsevier). The search terms were “Good Nursing Care Scale”, “Good Perioperative Nursing Care Scale”, or “Child Care Quality at Hospital”. The search was performed widely and all text fields including the specific search term were screened. Searches were also performed utilising a list of researchers (handled by the instrument copyright holder) who have been granted permission to use the scale. A manual search of the references of the included studies was also made.

The inclusion criteria for empirical studies were as follows: (1) The scale was used for data collection amongst patients; (2) the study was published in English or Finnish; (3) in a peer‐reviewed journal or/and as a summary of a PhD thesis; and (4) it was available as a full‐text paper. Studies including nurses as informants were excluded. The database searches were limited to English or Finnish peer‐reviewed publications when possible.

### 3.2 Screening and Quality Appraisal

**FIGURE 1:**
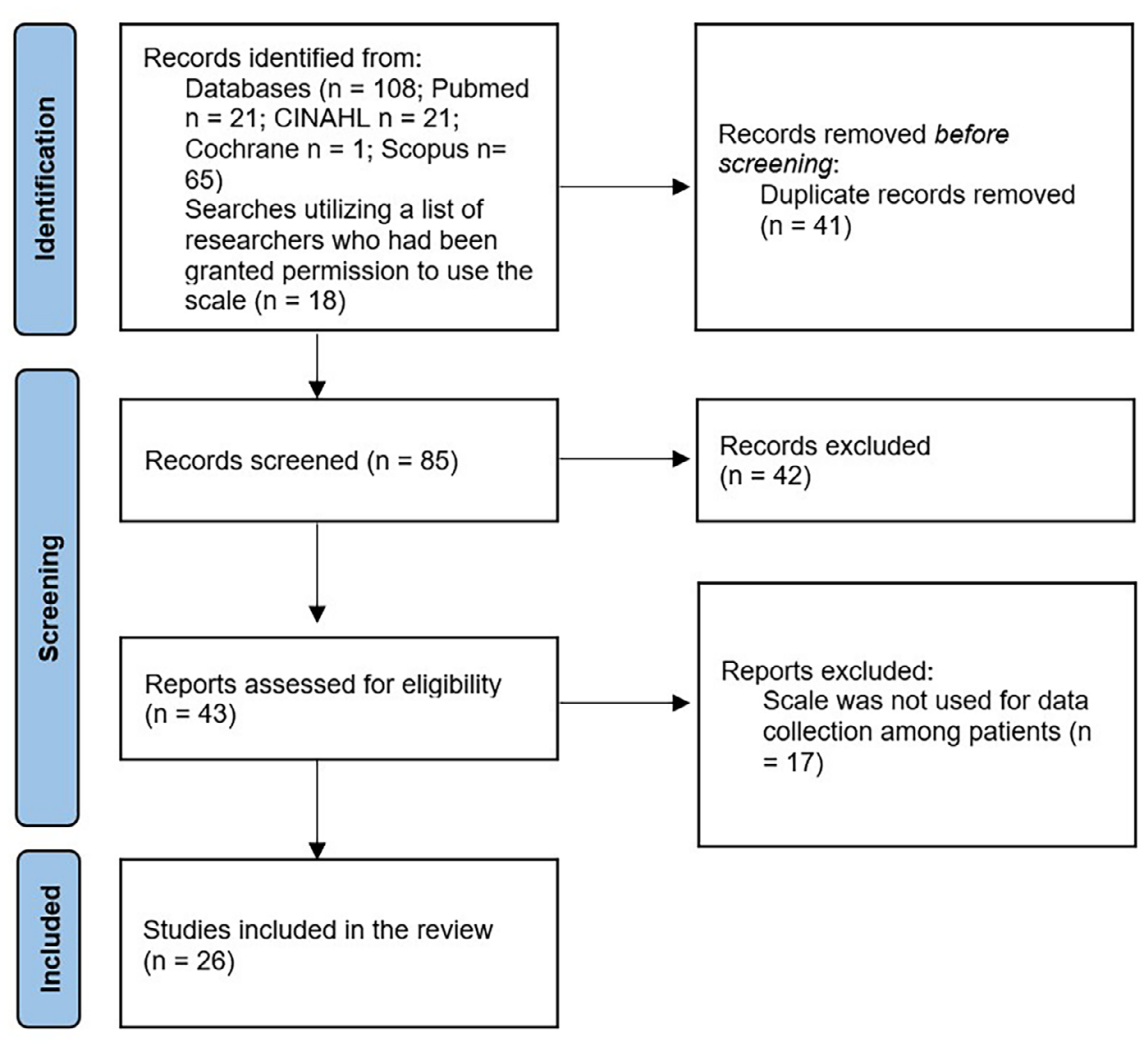
Flow diagram of the data selection process. [Colour figure can be viewed at wileyonlinelibrary.com]

The screening and retrieval process was conducted within the research team (Figure 1). Overall, 126 publications were identified. Duplications were identified with Zotero Reference Management Software. The title and abstract were screened, and the full‐text reports were assessed for eligibility. Finally, 26 studies met the inclusion criteria. No automation tools were used in the process.

The quality appraisal was conducted using a tool constructed for the purposes of this systematic review, as a specific quality appraisal tool focusing on instrument development or validation was not available. The tool was based on COSMIN reporting guidelines for studies on measurement properties of patient‐reported outcome measures (Gagnier et al. 2021, Appendix S3). General reporting recommendations for studies on measurement properties (Gagnier et al. 2021) were modified to serve as a quality appraisal tool, including a total of 35 items. Based on the original recommendation (Gagnier et al. 2021), the items were divided to focus on title (3 items), abstract (7 items), general methods (8 items), general results (3 items), discussion (6 items), conclusions (1 item), and other sections including conflicts of interest (1 item). Each item was evaluated using a three‐point scale: “yes” (indicating reporting of the item in the article), “no” (the item was not reported), and “unclear” (the item was partly reported or reporting was unclear).

### 3.3 Data Abstraction and Synthesis

One researcher (TM) extracted descriptive details from the publications (as seen in Tables 2, 3, 4, 5 and Appendix S4), and the research team analysed the data. If data of interest was missing, no assumptions were made. To clarify the result tables, we coded the results with numbers, and those studies that were conducted with the Good Perioperative Nursing Care Scale (GPNCS) or Child Care Quality at Hospital (CCQH) Scale are identified with the same number and separated with a different lower‐case letter. Also, the studies that used the same data collected with the GNCS were identified with the same code number and differentiated with a lower‐case letter. The synthesis was performed by using descriptive statistics (frequency, mean, standard deviation), narrative analysis and evaluation of psychometric properties. The summary of the means was calculated by multiplying the mean from each subscale by the number of observations in that subscale, summing these results, and then dividing the sum by the total number of observations (see Table 3 and Appendix S4). Narrative analysis focused on understanding the use of the GNCS and the level of quality of care and the characteristics associating with the quality of care.

The psychometric properties of the GNCS were analysed using generally agreed methodological criteria (Streiner and Norman 2003). The criteria covered the assessment of reliability (test–retest/stability, internal consistency, responsiveness) and validity (face, content, construct, predictive, criterion, discriminant, convergent, divergent). The analysis was done on descriptive level where reporting of reliability and validity testing was considered sufficient.

## 4 Results

### 4.1 Introduction to the Good Nursing Care Scale

**FIGURE 2:**
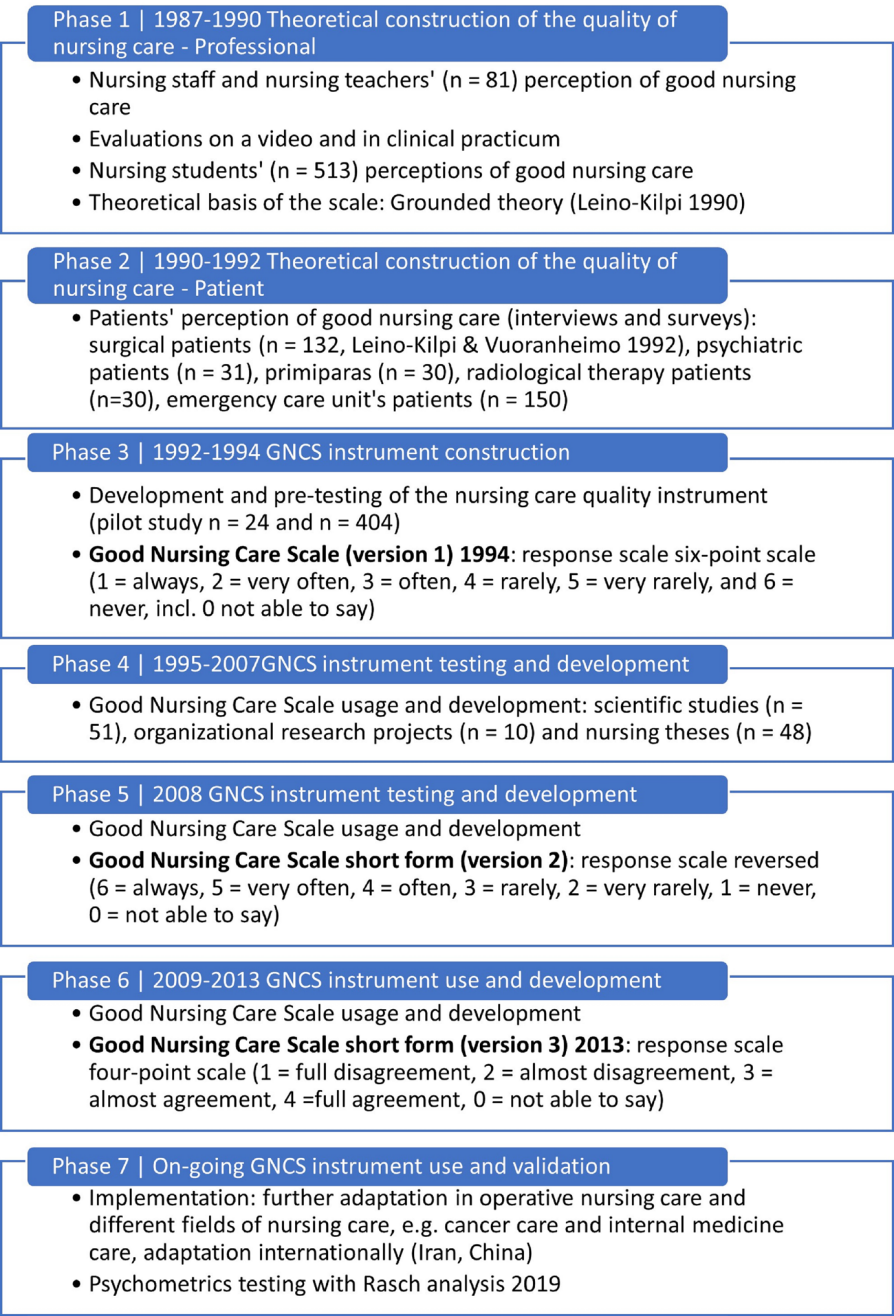
The development phases of the Good Nursing Care Scale (GNCS). [Colour figure can be viewed at wileyonlinelibrary.com]

The GNCS was developed in the early 1990s (Figure 2) following a systematic process. In the original study (Phase 1), the respondents described their view of good nursing care, evaluated the quality of nursing care action on a video, and the activities of nursing students in clinical practicum were observed (*n* = 81, Leino‐Kilpi 1990, 1992), establishing the theoretical construction of the quality of nursing care. After that (Phase 2), the theoretical construction was strengthened by interviews amongst different groups of patients, such as surgical (*n* = 132), psychiatric (*n* = 31), and maternity patients (*n* = 30), those undergoing radiological investigations (*n* = 30), and those in emergency care (*n* = 150) (Leino‐Kilpi and Vuorenheimo 1993).

Based on theoretical construction, the first version of the GNCS for patients was created in 1994 (Phase 3, Leino‐Kilpi and Vuorenheimo 1994) and piloted amongst hospital patients (*n* = 24 and *n* = 404). This first version included 116 items, and the structure of the scale was confirmed by a factor analysis (Leino‐Kilpi et al. 1994). After that, during the years 1995 and 2007 (Phase 4), the scale was tested several times to confirm content and structural validity and identify redundant items: scientific studies (*n* = 51), research projects of the organisations (*n* = 10), and nursing students' master theses at university (*n* = 48). Summative evaluation combining information from these individual studies and projects resulted in a shortened version of the scale (54 items) in 2008 (Phase 5). After this, systematic development work aimed at achieving a short, precise, and cohesive version of the GNCS continued, and in 2013, the 40 item‐scale was launched (Phase 6).

In addition to the original GNCS, there are also two versions of the GNCS developed for specific nursing areas. The GPNCS (54 items) was developed to evaluate the quality of perioperative nursing care (Leinonen et al. 2001; Leinonen 2002), and the CCQH (58 items) was developed to evaluate the quality of paediatric nursing care (Pelander, Leino‐Kilpi, and Katajisto 2007, 2009; Pelander 2008).

Theoretical construction of GNCS is based on nursing care as human action, defined by using grounded theory amongst nurses (*n* = 290) and nursing teachers (*n* = 223, Leino‐Kilpi 1990, 1991, 1992). Generally, human action theory is a broad perspective on human action (see, e.g., Mead 1934; Parsons and Shils 1951; Goldman 2016; Eyster, Satterfield, and Chan 2022), including six elements, i.e., the actor and actor's characteristics, activities and the nature of them, and preconditions and aims of the activities (Leino‐Kilpi 1990, 1992). The GNCS includes the summative factors of characteristics of the nurse/professional (actor), nursing actions (activities), prerequisites for nursing care (prerequisites), nursing environment (environment), progress of nursing process (procedure), patient's empowerment strategies (outcomes), and collaboration with family members (outcomes). It represents the manifestation of high quality, i.e., all subcategories and items are assumed to express desired quality of care. The theoretical construction of the scale has remained constant even though the number of items has decreased from 116 (1994) to 54 (2008) and then again to 40 (2013) (Table 1).

**TABLE 1:** Good Nursing Care Scale for Patients: Structure and content of different versions.

Quality categories	What	No. of items in different versions of Good Nursing Care Scale						The category includes (for example)
		No 11994	No 22008	No 32013	Modified versions			
					GPNCS 54‐item version	GPNCS 34‐item version	CCQH	
Nursing staff characteristics	Actor	12	6	5	8	4	11	Honest, careful, willingness to serve
Nursing care activities	Activities	40	10	6	35	^a^	28	Professional manner, educational, encouragement, mental support
Preconditions for care	Prerequisites	14	7	5	2	^a^	0	Nurses' knowledge and skills are up to date, evidence‐based knowledge and patients' good is a priority
Nursing environment	Environment	16	8	5	7	5	19	Safety, aseptics, identity checks and personal integrity
Proceeding of the nursing process	Procedure	20	10	6	2	2	0	Fluency, collaboration/integration between different care organisations, discharge information
Patients' empowerment strategies	Outcomes	14	13	7	0	0	0	Patients' opinions are taken into account, patients are aware of the treatment and financial costs and benefits
Collaboration with family members	Social setting	0	0	6	0	0	0	Family members are informed, heard, supported and participate in care
Scales in different versions of the Good Nursing Care Scale		1–6	1–5	1–4	1–5	1–5	1–3/1–4 1–5 With face pictures	

Abbreviations: CCQH, child care quality at hospital Pelander; GPNCS, good perioperative nursing care scale Leinonen.

^a^ The names of the sum variables were abridged and some of them revised not aligning with the original structure of the Good Nursing Care Scale.

In the original version (1994), the response scale for evaluating the quality of care was a six‐point scale, expressing the frequency of the content of the item. Based on this, the frequency of the appearance of the elements of quality indicated the degree of quality; less frequent means higher quality. In version two (2008), this scale was reversed for logical reasons. Based on this, the indication of quality was more logical: more frequent indicated higher quality. The aim of the six‐point scale was to increase the distribution and validity of the results of the new scale. Through testing, the scale evolved into a five‐point scale, and instead of frequency, the respondents expressed the extent to which they agreed with the statement. In version three (2013), the scale was shortened to four points. The four‐point scale was agreed on so as to eliminate the neutral option in the middle of the measurement scale in order to support respondents to express a clear position on quality. The mean quality score given is interpreted as follows (GNCS, version 3): mean 1.0–1.5 = very low quality of care, 1.6–2.0 = low quality of care, 2.1–2.5 = fairly low quality of care, 2.6–3.0 = fairly high quality of care, 3.1–3.5 = high quality of care, and 3.6–4.0 = very high quality of care.

Use of the GNCS requires permission from the copyright holder, and the researchers who have been granted permission are expected to send their results (report/summary) to the copyright holder.

### 4.2 Study Characteristics

In total, 26 full‐text studies and summaries of PhD thesis were included in the analysis (Table 2, 1–13). The GNCS has been used in 10 countries in different areas of nursing. In addition to Finland, studies using the scale have been conducted in China, Denmark, Estonia, Iran, Italy, Lithuania, Portugal, Sweden, and Turkey. The nursing areas whose quality has been evaluated are various. The reported specified areas are cancer care (*n* = 134), internal medicine nursing care (*n* = 197–200), paediatric nursing care (*n* = 57–692), perioperative nursing care (*n* = 90–874), postnatal nursing care (*n* = 869), and postoperative nursing care (*n* = 132–1208). Related to GNCS, testing of the instrument has been both national (1b, 4, 5b, 6a) and international (1d, 2, 3, 7, 8). Furthermore, versions have been established for perioperative nursing care (GPNCS; 1a, 1b, 1c) and for child care (CCQH; 5a, 5b, 5c). Since 2013, implementation and evaluation of the instrument have taken place both in Finland and in other countries. These include Iran amongst cardiac patients (11a, 11b), Italy amongst paediatric patients (5d), and Turkey amongst perioperative patients (1d). In Finland, the connection of quality with family members (10a) and patient education (10b) or postoperative complications (12) has been investigated. Currently, there are 10 different language versions of the GNCS.

**TABLE 2:** An overview of empirical studies using the Good Nursing Care Scale for patients (*n* = 26).

Code in the analysis Author(s), year	Country	Area of nursing care	*n* (adult patients if not stated otherwise)	Design	Purpose/aim
1a Leinonen et al. (2001)^d^	Finland	Perioperative	874		“to find out how surgical hospital patients perceived the quality of perioperative care they received in an operating department and in the recovery room”
1b Leinonen (2002)^d^	Finland				“to describe the quality of perioperative care as evaluated by patients and perioperative nurses” “to develop measurement tool that patients can use when assessing their perioperative care”
1c Leinonen et al. (2003)^d^	Finland			Descriptive	“to explore and compare patients' and their nurses' perceptions of the quality of perioperative care and to identify possible differences in these perception”
1d Donmez and Ozbayır (2011)^d^	Turkey Turkish translation of the GPNCS		346	Cross‐sectional survey	“to test the validity and reliability of the Turkish version of Good Perioperative Nursing Care Scale (GPNCS) for nurses and patients”
1e Hertel‐Joergensen, Abrahamsen, and Jensen (2018)^d^	Denmark Danish translation of the GPNCS		215	Cross‐sectional survey	“to test the psychometric validity of the GPNCS, a self‐administered questionnaire, following translation and adaptation”
1f İbrahimoğlu et al. (2022)^d^	Turkey Turkish translation of the GPNCS		90	Descriptive	“to examine the relationship between perioperative care quality and postoperative comfort level of patients undergoing hip replacement surgery”
2 Rehnström et al. (2003)^a^	Sweden Swedish translation of the GNCS	Postoperative	447		“to adapt the instrument ‘Good Nursing Care Scale for Patients’ to Swedish conditions as a measure of patients' satisfaction, as well as estimating its reliability and validity”
3 Kalam‐Salminen (2005)^a^	Estonia, Finland	Postnatal	489 (mothers) 380 (fathers)		“to assess client‐centered quality in postnatal wards from the perspective of mothers, fathers as well as nursing personnel, and search for quality‐related factors and ways for quality development”
4 Ruotsalainen (2006)^a^	Finland	Internal medicine nursing care	197		“to evaluate the quality of nursing care of internal medicine patients and describe the patient's participation in the evaluation of the quality of care”
5a Pelander, Leino‐Kilpi, and Katajisto (2007)^e^	Finland	Paediatric	388 (children)	Descriptive	“to evaluate the quality of paediatric nursing care as perceived by Finnish children aged 7 to 11 by using an instrument (Child Care Quality at Hospital) developed on the basis of children's expectations of the quality of care”
5b Pelander (2008)^e^	Finland		445 (children)	Three‐phase study descriptive and explorative	“to improve the quality of paediatric nursing in hospital”
5c Pelander, Leino‐Kilpi, and Katajisto (2009)^e^	Finland		41 (children, pilot test I), 16 (children, pilot test II)	Descriptive and explorative	“report of the development and psychometric evaluation of the Child Care Quality at Hospital (CCQH) instrument”
5d Comparcini et al. (2018)^e^	Italy Italian translation of the CCQH		692 (children)	Multicenter cross‐sectional study	To explore “children's perceptions about the quality of nursing care and the determinants of their evaluations according to different categories of children's ages”
5e Loureiro, Araújo, and Charepe (2019)^e^	Portugal Portuguese translation of the CCQH		252 (children)		“adapt and validate the instrument CCQH to assess the quality of nursing care of hospitalised children for the Portuguese language of Portugal”
6a Siekkinen et al. (2008)^a^	Finland	Cancer care	134	Descriptive	“to describe patients' experiences of the quality of care received at a radiotherapy centre”
6b Siekkinen (2014)^a^	Finland			Descriptive, longitudinal and experimental randomised controlled trial	“to develop quality of radiotherapy care by the e‐Feedback knowledge of radiotherapy ‐intervention (e‐Re‐Know)”
7 Zhao, Akkadechanunt, and Xue (2009)^b^	China Chinese translation of modified GNCS (Perception of Quality Nursing Care Scale)	Various	383	Descriptive, comparative study	“to explore and compare nurses and patients perceptions of quality nursing care”
8 Istomina (2011)^b^	Lithuania Lithuanian translation of GNCS	Postoperative	80 (adaptation), 1208		“to evaluate the quality of abdominal surgical nursing care and the factors related to it as perceived by patients following abdominal operations and by surgical nurses”
9 Leino‐Kilpi et al. (2015)^b^	Finland	Various	226		“to evaluate and analyse the connection between the level of quality of nursing care and knowledge received by patients”
10a Leino‐Kilpi et al. (2016)^c^	Finland	Postoperative	481	Cross‐sectional descriptive survey study	“to describe the participation of family members in the care of Finnish adult surgical patients and the connection of the participation with the quality of patient care as perceived by surgical patients”
10b Gröndahl et al. (2019)^c^	Finland		480	Cross‐sectional descriptive correlational stud	“to analyse the relationship between patient education and the quality of surgical nursing care as perceived by patients”
10c Stolt et al. (2019)^c^	Finland		476	Explorative cross‐sectional study design	“to analyse the psychometric properties of the Good Nursing Care Scale (GNCS) in a sample of surgical patients and nurses”
11a Bahrami et al. (2018)^b^	Iran Persian translation of GNCS	Internal medicine nursing care	200	Cross‐sectional study	“to validate the Persian version of GNCS‐P”
11b Bahrami et al. (2020)^b^	Iran Persian translation of GNCS			Cross‐sectional study	“to explore cardiac patients' perception of a good nursing care”
12 Saarinen et al. (2020)^c^	Finland	Postoperative	323	Correlation cross‐sectional study	“to study if patient‐related factors are associated with patient‐evaluated quality of care in surgery” and “to examine if there is an association with postoperative complications and patient‐evaluated low quality of care”
13 Esmalizadeh et al. (2023)^c^	Iran Persian translation of GNCS	Various	200		“to translate and determine the psychometric properties of the Persian version of the “Good Nursing Care Scale” (GNCS‐P)”

^a^ Version 1 of the scale.

^b^ Version 2 of the scale.

^c^ Version 3 of the scale.

^d^ The Good Perioperative Nursing Care Scale (GPNCS).

^e^ Child Care Quality at Hospital Scale (CCQH).

The studies had been given ethical approval according to their national standards; in most countries, this is required due to the sample consisting of patients.

Based on quality appraisal, the quality of reporting was generally at a good level (Appendices [Link], [Link], [Link], [Link]). Quality appraisal of the included studies using a modified tool based on Gagnier et al. (2021). Titles, abstracts, and introductions were predominantly well reported, including the description of measurement properties, state of knowledge and rationale for the study. However, in the methods section, the main deficits were reporting study design, sample size calculation, handling of missing data, and post hoc analyses. In the discussion sections, discussion on instrument changes was limited, and conflicts of interest were seldom reported.

### 4.3 Level of the Patient‐Centered Quality of Nursing Care

Overall, patients' assessment of the quality of nursing care, assessed with GNCS, was reported to be at high level (1f, 6a, 6b, 9, 10a, 10b, 11b, 12, Table 3 and Appendix S4). Slight variation between subscales was identified on the assessed level of the quality of that dimension. In most of the studies, the level of the dimensions of nursing staff characteristics (1a, 1b, 1c, 3, 6a, 6b, 7, 8, 9, 10a, 10b, 11b, 12), nursing care activities (4, 6a, 6b, 7, 9, 10a, 10b, 11b, 12), preconditions for care (3, 7, 8, 9, 10a, 10b, 12), nursing environment (3, 6a, 6b, 7, 8, 9, 10a, 10b, 11b, 12), and proceeding of the nursing process (1a, 1c, 4, 6a, 6b, 7, 8, 9, 10a, 10b, 12) varied from high to very high. However, there were also fairly high levels in the dimensions of nursing staff characteristics (4), preconditions for care (4, 11b), nursing environment (4), and proceeding of the nursing process (11b). No low levels were reported in the above‐mentioned dimensions. However, fairly low levels were found in the dimensions of Patients' empowerment strategies and Collaboration with family members (11b). Altogether, the dimensions of patients' empowerment strategies (9, 10a, 10b, 12) and collaboration with family members (4, 7, 8, 10a, 10b, 11b, 12) were still described to be at a high to very high level in other studies. No clear difference between countries was identified, although there were no comparable data between the countries.

**TABLE 3:** The level of quality of nursing care in the studies using Good Nursing Care Scale: summary of the means in different versions.

Version of the Good Nursing Care Scale	Studies	Nursing staff characteristics			Nursing care activities			Preconditions for care			Nursing environment			Proceeding of the nursing process			Patients' empowerment strategies			Collaboration with family members			Total		
		M	SD	*n*	M	SD	*n*	M	SD	*n*	M	SD	*n*	M	SD	*n*	M	SD	*n*	M	SD	*n*	M	SD	*n*
The Good Perioperative Nursing Care Scale (GPNCS) Leinonen, 54‐item version, scale 1–5	1a, 1b, 1c	4.82	0.32	778	4.48	0.48	377	4.61	0.58	675	4.78	0.38	708	4.64	0.61	743							4.7^a^		
The GPNCS Leinonen, 34‐item version, scale 32–160 (sum of the results)	1e, 1f	22.09	2.12	305	101.14	12.96	305				18.57	1.86	305	8.79	1.43	90							143.06	14.68	305
Child Care Quality at Hospital (CCQH) Pelander, scale 1–3 in “Nursing staff characteristics” and “Nursing care activities”, scale 1–4 in “Nursing environment”	5a, 5b	2.66	0.24	388	2.48	0.29	385				3.18	0.44													
CCQH Pelander, scale 1–5	5d	3.79	1.07	692	3.03	0.96	692				2.78	0.64	692										3.96	0.93	692
Version 1 from 1994, scale 1–6 (reversed to the others)	3, 4	3.46	0.41	934	1.97	0.78	100	3.30	0.50	798	3.44	0.41	889	1.34	0.88					1.47	2.03				
Version 1 from 1994, scale 1–4 (reversed to the others)	6a, 6b	1.15	0.25	131	1.38	0.35	130				1.42	0.26	129	1.37	0.34	129							1.33^b^		
Version 2.0 from 2008, scale 1–6	8, 9	5.44	0.74	1289	5.18	0.87	222	5.20	0.91	1276	5.37	0.91	1279	4.57	0.96	1188	5.17	0.85	210	4.55	1.36	679	5.29^c^	0.65	
Version 2.1 from 2008, scale 1–5	7, 11b	3.84	0.69	583	3.73	0.69	583	3.67	0.80	583	3.85	0.69	583	3.68	0.73	583	2.37	1.02	200	3.56	0.83	283	2.81^d^	0.75	200
Version 3 from 2013, scale 1–4	10a, 10b, 12	3.81	0.36	1209	3.68	0.43	1203	3.70	0.44	1181	3.84	0.30	1205	3.66	0.41	1196	3.54	0.50	1176	2.98	0.91	852	3.50	0.34	1219

Abbreviations: M, mean; SD, standard deviation.

^a^ From 1a.

^b^ From 6b.

^c^ From 9.

^d^ From 11b.

### 4.4 Variables Associated With the Quality of Nursing Care

The GNCS has been used with varying background and other variables, depending on the aim of the study and the country. Altogether, 92 different potentially associated variables were used in the studies using the patient version of the GNCS. Statistically significant associations (significance level *p* < 0.05) were identified between the summative quality of the nursing care and 18 variables (Table 4). However, out of those 18 variables, five were inconsistent, showing statistical significance in some studies while being statistically insignificant in others: age (1f, 6a, 6b, 8, 9, 10b, 11b), educational background (6a, 6b, 10b, 11b), hospitalizations before the current one (10b, 11b), living arrangement (10b, 11b, 12), and work status (1f, 6a, 6b, 10b).

**TABLE 4:** Variables tested for the association with the quality of nursing care (available information presented).

	Variables (studies)
Statistically significant association with quality of the nursing care	Adequate information about hospital care (9, 10b) Age (6a, 6b, 8, 9) Educational background (6a, 6b) Experienced comfort after hip surgery (1f) Having prehospital information (9, 10b, 11b) Having surgery complications (12) Hospitalizations before the current one (11b) Information about the progress of the care and treatment (9, 10b) Living arrangement (12) Potential to select another hospital (12) Satisfaction with health care in current hospital (5d) Satisfaction with medical treatment during the hospitalisation (8) Satisfaction with nursing care during the hospitalisation (8) Satisfaction with health care (8) The hospital where the patient stayed (1f, 11b) Use of mobility assistive devices (1f) Well‐being at the time of the answer (own assessment) (9, 10b, 12) Work status (6a, 6b)
Statistically insignificant association with quality of the nursing care	Age (1f, 10b, 11b) BMI (1f) Charlson Comorbidity Index (12) Duration of the hospital stay (10b) Duration of the operation (1f) Duration of the stay in the recovery room (1f) Educational background (10b, 11b) Gender (1f, 10b, 11b, 12) Having chronic diseases (1f, 10b) Hospitalizations before the current one (10b) If relatives were involved in the treatment (10a) Living arrangement (10b, 11b) Marital status (1f) MET Index (12) Number of mobilizations on the second postoperative day (1f) Number of previous surgeries (1f) Occupation (11b) Pain intensity during hospital stay (1f) The procedure that was done (12) Whether the hospitalisation was elective or emergency (10b, 12) Work status (1f, 10b)

*Note:* Significance level *p* < 0.05; OBS! This analysis indicates the existing statistical association, not the direction of it.

### 4.5 Psychometric Properties of the GNCS

Psychometrics of the GNCS were tested diversely in the studies (Table 5). In validity evaluation, content (in 19 studies), construct (in 14), face (in 7), criterion (in 4), and item analysis (in 3) were reported. Content validity was examined by expert panels (1d, 1e, 2, 4, 5a, 5b, 5c, 7) and pilot testing (1a, 1b, 1c, 1d, 1e, 3, 4, 5a, 5b, 5c, 5d, 6a, 6b, 7, 8). Construct validity was tested using confirmatory factor analyses (1e, 3, 11a) and principal component analyses (1d, 5b, 5c, 5e, 8), in addition to some types of factor analyses that were not specified in the studies (1b, 2, 4, 6a, 6b). Fit indices were applied to confirm construct validity (11a, 13). Face validity was assessed by experts and participants (1e, 8, 11a, 13). However, discriminant, convergent, and divergent in validity were not evaluated in any studies.

**TABLE 5:** Psychometric properties tested of the Good Nursing Care Scale.

		Studies that tested psychometric properties	How the property was tested
Validity	Face	7 studies: 1d, 1e, 5d, 5e, 8, 11a, 13	Experts and participants (1e, 8, 11a, 13)
	Content	19 studies: 1a, 1b, 1c, 1d, 1e, 2, 3, 4, 5a, 5b, 5c, 5d, 5e, 6a, 6b, 7, 8, 11a, 13	Expert panels (1d, 1e, 2, 4, 5a, 5b, 5c, 7) Pilot testing (1a, 1b, 1c, 1d, 1e, 3, 4, 5a, 5b, 5c, 5d, 6a, 6b, 7, 8) Content validity index was also calculated indicating good content validity (5c, 11a, 13) When translated, affirmed by experts or with forward and back translation (1d, 1e, 2, 3, 5d, 5e, 7, 11a, 13)
	Construct	14 studies: 1b, 1d, 1e, 2, 3, 4, 5b, 5c, 5e, 6b, 8, 10c, 11a, 13	Confirmatory factor analyses (1e, 3, 11a) Principal component analyses (1d, 5b, 5c, 5e, 8) Factor analyses (1b, 2, 4, 6a, 6b) Fit indices (11a, 13)
	Criterion	4 studies: 1b, 3, 4, 13	Measurements were performed at five different locations at the same time (1b) The assessment of the quality of nursing care was concurrent in different hospital environments (3) Data were collected at two stages with partly the same scale, the results were concurrent, and hence, showed criterion validity (4) The data was collected with the Good Nursing Care Scale and a corresponding instrument, and the results of these were compared to evaluate the criterion validity (13)
	Mention of validity being previously reported	16 studies: 1b, 1e, 1f, 2, 4, 5b, 5d, 5e, 6b, 9, 10a, 10b, 10c, 11b, 12, 13	
Reliability	Internal consistency	24 studies: 1a, 1b, 1c, 1d, 1e, 1f, 2, 3, 4, 5a, 5b, 5c, 5d, 5e, 6a, 6b, 7, 8, 9, 10a, 10b, 10c, 11a, 13	
	Stability	6 studies: 1b, 1d, 1f, 6b, 11a, 13	Test–retest method (1b, 2, 6a, 6b, 11a, 13)
	Item analysis	3 studies: 2, 5b, 5c	Item‐to‐total correlations (2, 5b, 5c)
	Mention of reliability being previously reported	14 studies: 1b, 1f, 2, 4, 5b, 5d, 5e, 6a, 6b, 7, 10a, 10c, 11b, 13	
	No reporting of reliability	1 study: 12	
Ethics	Permission to use the instrument reported	8 studies: 1e, 5d, 6a, 6b, 7, 8, 11a, 13	

Regarding reliability, internal consistency of categories and/or whole instruments was most commonly evaluated (reported in 24 studies, Table 5) and indicated good consistency (Cronbach alphas 0.79–0.95, Table 6). In some categories, variation in consistency was higher than in others, but mostly on an acceptable level (Nunnally and Bernstein 1994). Moreover, test–retest method was utilised to confirm good stability (1b, 2, 6a, 6b, 11a, 13), and item‐to‐total correlations were used as an item analysis method (2, 5b, 5c). Responsiveness evaluating reliability was not in any studies.

**TABLE 6:** Consistency of the Good Nursing Care Scale in different studies (Cronbach's alpha).

Study	Nursing staff characteristics			Nursing activities			Preconditions for care			Nursing environment			Proceeding of the nursing process			Patients' empowerment strategies			Collaboration with family members			Total
	α	No of items	*n*	α	No of items	*n*	α	No of items	*n*	α	No of items	*n*	α	No of items	*n*	α	No of items	*n*	α	No of items	*n*	α
1a	0.86	8	778	0.79	35	377	0.66	2	675	0.84	7	708	0.14	2	743							
1c	0.86	8	778	0.79	35	377	0.66	2	675	0.84	7	708	0.14	2	743							
1d																						0.93
1e	0.87	5	215							0.82	4	215										0.92
1f	0.90	4	90							0.89	4	90	0.78	2	90							0.92
2	0.95	32	447	0.90	23	447																0.80
3	0.89	11	178				0.91	14	120	0.90	17	164										
	0.91	11	115				0.96	14	88	0.94	17	106										
	0.88	11	286				0.90	14	262	0.87	17	272										
	0.88	11	255				0.90	14	228	0.90	17	247										
4	0.92	29	100										0.78	21	100							
5a	0.38–0.56	11 5	388	0.82–0.81	28 25	388				0.76	19	388										
5b				0.76	25	41				0.55	23	41										
				0.57	29	16				0.58	19	16										
	0.38–0.56	11 5	388	0.82–0.81	28 25	388				0.76	19	388										
5c	0.56	5	388	0.81	25	388				0.76	19	388										
5d	0.69–0.79	5	692	0.87–0.94	25	692				0.74–0.81	19	692										0.89–0.94
5e	0.75	5	252																			
6a	0.79	7	131	0.84	19	130				0.52	10	129	0.39	5	129							0.79
6b																						0.79
7																						0.81
8	0.926	14	80	0.921	19	80	0.959	8	80	0.828	2	80	0.688	10	80				0.964	12	80	0.881
	0.96	14	1063	0.94	19	1062	0.94	8	1059	0.71	2	1056	0.82	10	969				0.97	12	679	
9	0.927	6	226	0.924	10	222	0.918	7	217	0.689	8	223	0.832	10	219	0.950	13	210				
10a				0.80						0.66									0.94			0.94
10b	0.773	5	470	0.839	6	467	0.797	5	453	0.660	5	466	0.709	6	459	0.842	7	447	0.940	6	316	0.940
10c																						0.81
11a	0.87	5	200	0.88	6	200	0.79	5	200	0.85	5	200	0.79	6	200	0.84	7	200	0.92	6	200	0.95
13	0.892	5	200	0.931	6	200	0.911	5	200	0.932	5	200	0.947	6	200	0.885	7	200	0.900	6	200	0.865

Abbreviation: α, Cronbach's alpha coefficient.

Psychometric properties of the GNCS have also been evaluated with Rasch analysis using a sample of surgical patients (*n* = 480, Stolt et al. 2019). The GNCS demonstrated good unidimensionality (64.4%), person separation, and the Rasch equivalent Cronbach's alpha (0.81), thus supporting the reliability of the GNCS.

In summary, validity and reliability evaluation and reporting were rather systematic in the studies and mainly indicate sufficient level. The variations between countries are not large, supporting the international use of the GNCS. Referring to ethical quality, permission to use the instrument was reported in only seven of the studies (1e, 5d, 6a, 6b, 7, 8, 11a, 13).

## 5 Discussion

In this systematic review, we have described the GNCS (Leino‐Kilpi 1990), its development and use over the years in patient‐centered quality evaluation, aiming to support researchers, practitioners, leaders, and educators to use validated instruments. Even with progress in patient‐centered quality evaluation, there still is a need for improvement (Wong, Mavondo, and Fisher 2020).

The theoretical base of the GNCS lies on the fundamental theory of human action (Parsons and Shils 1951; Mead 1934), emphasising the elements of actor, activities, preconditions, and outcomes of action, specified in subcategories and updated in empowerment strategies of patients and collaboration with patients' significant others. As fundamental in nursing, this theoretical structure stands the test of time, being relevant for the future as well.

The results of the studies using the GNCS indicate a rather high quality of nursing care as evaluated by patients (see Table 3 and Appendix S4). It has been indicated that patients tend to evaluate quality of care positively because of their loyalty to the health care service provider (Keller et al. 2014), or the reason can be social desirability (Van de Mortel 2008) and evaluation apprehension (Keller et al. 2014). On the contrary, the negative halo effect can be present (Burke et al. 2020), meaning that negative experiences in some parts of the care process lead to negative evaluations of the entire process. In addition, patients' dissatisfaction with their care can transfer to their evaluations on other occasions. In the testing of the GNCS, in order to obtain trustworthy results, we have used different measurement scales and have also interviewed patients without much change in the level of quality. In the future, however, observations of nursing episodes as well as group‐evaluations of patients could be added.

Based on GNCS, some areas of quality need further consideration. These are educational activities of nurses (Siekkinen 2014; Siekkinen et al. 2008; Leinonen 2002; Leinonen et al. 2001, also identified by Gishu, Weldetsadik, and Tekleab 2019; Karaca and Durna 2019; Forsberg et al. 2015; Fröjd et al. 2011), proceeding of the nursing process (Leino‐Kilpi et al. 2015; Istomina 2011; Ruotsalainen 2006), which is also seen in coordination (Naef, Ernst, and Petry 2019) and continuity of care (Törnvall and Wilhelmsson 2010), patient participation in care, support for patients' initiatives and patients' encouragement (Gröndahl et al. 2019; Siekkinen et al. 2008; Ruotsalainen 2006; Leinonen et al. 2001, also identified by Alsyouf et al. 2018; Forsberg et al. 2015; Fröjd et al. 2011; Törnvall and Wilhelmsson 2010), as well as collaboration and planning of care with family members (Saarinen et al. 2020; Gröndahl et al. 2019; Siekkinen 2014; Istomina 2011; Siekkinen et al. 2008; Ruotsalainen 2006).

The quality category of empowerment strategies of patients deserves a specific mention. This category has high importance for the future, emphasising the empowerment of citizens and health service users (European Union 2021; EMPATHiE Consortium 2014; International Alliance of Patients' Organizations 2012). This category was added later to the GNCS to describe patients' own responsibility in care and is identified as one needing improvement (Bahrami et al. 2020; Gröndahl et al. 2019). The connection between quality of care and empowerment of patients has been identified to be positive (Gröndahl et al. 2019). However, this connection requires further research even though there are not so many evaluative instruments on empowerment (Pekonen et al. 2019).

Several factors seem to be connected with the quality of patient‐centered nursing care. One central factor is the information and knowledge of patients. Improvement in this quality area is clearly needed, including the diagnostics of patients' knowledge expectations and instrumentation for that (see Leino‐Kilpi et al. 2015). Register‐based data of patients, added to quality evaluation, would be relevant adding for taking the social determinants of health under consideration.

Quality is also connected with patients' health and well‐being, with higher evaluations being connected with higher quality (Saarinen et al. 2020; Gröndahl et al. 2019; Leino‐Kilpi et al. 2015), in line with other studies (Karaca and Durna 2019; Suhonen et al. 2018; Wilde et al. 1995), and with complications during the care process (Saarinen et al. 2020). General satisfaction with care has been reported, somewhat inconsistently, to be connected with the categories of GNCS (Comparcini et al. 2018; Istomina 2011), in contrast with other studies (Al‐Awamreh and Suliman 2019; Hatami et al. 2018). There are also other unsystematic results related to different factors. In the future, a systematic, comprehensive literature review about these factors is needed.

The GNCS shows reliability and validity as demonstrated by classical and modern test theory approaches; it has been developed using a systematic, long‐term process and has been validated in different languages. Thus, the GNCS is a usable instrument for future patient‐oriented quality evaluation. In its current 40‐item format, it can be used in connection with other instruments and for measuring outcomes of interventions. However, the role of the patient in quality evaluations could be defined in a more systematic way, for supporting their important contribution in quality assurance of health services.

Currently, quality instruments are mainly used in single studies or in studies with different groups of patients with a large amount of background factors, and this is also the case with the GNCS. It has not, however, been systematically evaluated in connection with other quality instruments. A large, multidimensional analysis of the quality of nursing care using several valid instruments could identify best practices between countries and groups of patients. This would be supported by the emphasis on a patient‐centered approach seen in most of the strategic papers (OECD 2022; European Union 2021; WHO et al. 2018). In the GNCS, different categories could also be used separately for the purposes of evaluation. For example, the characteristics of the actor could be relevant criteria for evaluation of individual nurses. Using the scale for different purposes would express willingness for sustainable actions.

In this paper, there were some limitations. An extensive literature search to identify English‐ and Finnish‐language studies was performed in four international scientific databases, supplemented with manual searches. The GNCS may also have been used in theses of students at different levels as well as an outcome measurement in intervention studies not reported here. The language limitation and the databases searched may also have limited the number of studies. The research team evaluated all potential articles, first on the title and abstract level and then on the full‐text level, resulting in an agreement on the studies included in the systematic review. The analysis was done based on an analysis plan that was created in advance. Data analysis remained on a descriptive level. The heterogeneous nature of the research did not allow to conduct a meta‐analysis, for example.

## 6 Conclusions

In this paper, we have concentrated on the instrumentation of patient‐centered quality evaluation measured with the GNCS. The GNCS has been systematically developed and tested since the 1990s in several languages, countries, and nursing areas.

Based on its theoretical content and psychometrics, the GNCS is relevant to be used in the future. However, there is always a need for continuous, systematic process evaluation. In this evaluation, new data, for example, narrative or register‐based data, would be fruitful.

The GNCS has mainly been used for research purposes. In the future, the use of the scale would benefit from other purposes for systematic multidimensional quality analysis. Furthermore, the scale could be implemented internationally for supporting the empowerment of the health service users.

## Author Contributions

H.L.‐K. study planning and design. T.M., M.S., J.K. and H.L.‐K. data collection, data analysis, manuscript preparation and writing, reviewing, and editing. All authors read and approved the final manuscript.

## Conflicts of Interest

The authors declare no conflicts of interest.

## Supporting information


Appendix S1

Appendix S2

Appendix S3

Appendix S4

## Data Availability

The data that supports the findings of this study are available in the Supporting Information of this article.
