# Supplementary material for: Introduction and Systematic Review of the Good Nursing Care Scale
Source: J Clin Nurs. 2024 Oct 11;34(1):5–23. doi: 10.1111/jocn.17486 (PMC11655425; doi:10.1111/jocn.17486)
Supplement: Supplementary file 3 — Appendix S3 [file JOCN-34-5-s001.docx]

Appendix S3. Quality appraisal of the included studies using modified tool based on Gagnier et al. 2021.

| Item/Reference | Bahrami et al. 2018 | Bahrami et al. 2020 | Comparchini et al. 2018 | Donmez & Ozbayri 2010 | Esmalizadeh et al. 2022 | Gröndahl et al. 2019 | Hertel-Joergensen et al. 2018 | Ibrahimoglu et al. 2022 | Leino-Kilpi et al. 2015 | Leino-Kilpi et al. 2016 | Leinonen et al. 2001 | Leinonen et al. 2003 | Loureiro et al. 2019 | Pelander et al. 2007 | Pelander et al. 2009 | Rehnström et al. 2003 | Saarinen et al. 20202 | Siekkinen et al. 2008 | Stolt et al. 2019 | Zhao et al. 2008 |
| --- | --- | --- | --- | --- | --- | --- | --- | --- | --- | --- | --- | --- | --- | --- | --- | --- | --- | --- | --- | --- |
| T1: Patient Reported Outcome Measure (PROM) | y | y | y | y | y | y | y | y | y | n | y | n | y | y | y | y | n | n | y | n |
| T2: Measurement Property (MP) | uc | y | y | y | y | y | y | y | uc | y | y | y | y | y | y | y | y | y | y | y |
| T3: Study sample | y | y | y | y | n | y | y | y | y | y | y | y | n | y | y | y | y | y | n | y |
| A1: PROM | y | y | y | y | y | y | y | y | y | y | y | n | y | y | y | y | y | y | y | y |
| A2: Measurement Property | y | y | y | y | y | y | y | y | y | y | y | y | y | y | y | y | y | y | y | y |
| A3: Design | y | y | y | y | y | y | y | y | y | y | n | n | y | y | n | n | y | n | y | y |
| A4: Sample | y | y | y | uc | y | y | y | y | y | y | y | y | y | y | y | y | y | y | y | y |
| A5: Methods | y | y | y | y | y | y | y | y | n | y | y | y | y | y | y | y | y | y | y | y |
| A6: Results | y | y | y | y | y | y | y | y | y | y | y | y | y | y | y | y | y | y | y | y |
| A7: Discussion/conclusion | y | y | y | y | y | y | y | y | y | y | y | y | y | y | y | y | y | y | y | y |
| I1: Name and describe the PROM of interest | y | y | y | y | y | y | y | y | y | y | y | y | y | y | y | y | y | y | y | y |
| I2: Target population | y | y | y | y | y | y | y | y | y | y | y | y | y | y | y | y | y | y | y | y |
| I3: Citation for the original development of the PROM | y | y | y | y | y | y | y | y | y | y | y | y | y | y | y | y | y | y | y | y |
| I4: State of Knowledge & Rationale | y | y | y | y | y | y | y | y | y | y | y | y | y | y | y | y | y | y | y | y |
| I5: Definitions | y | y | y | y | y | y | y | n | y | y | y | y | y | y | y | y | y | y | y | y |
| I6: Objectives and Hypotheses | y | y | y | y | y | y | y | y | y | y | y | y | y | y | y | y | y | y | y | y |
| GM1: Study Design | y | y | y | y | y | y | y | y | y | y | n | y | y | n | n | n | y | n | y | y |
| GM2: Participants | y | y | y | y | y | y | y | y | y | y | y | y | y | y | y | y | y | y | y | y |
| GM3: PROM administratio | y | y | y | y | y | y | y | y | y | y | y | y | y | y | y | y | y | y | y | y |
| GM4: Data collection procedures | y | y | y | y | y | y | y | y | y | y | y | y | y | y | y | y | y | y | y | y |
| GM5: Power/sample size calculation | n | y | n | n | y | n | n | y | uc | uc | y | y | y | n | n | n | n | n | n | n |
| GM6: Statistical analyses | y | y | y | y | y | n | y | y | y | y | y | y | y | y | y | y | y | y | y | y |
| GM7: Missing data | n | n | n | n | n | n | n | n | n | n | n | n | n | n | n | y | n | n | n | n |
| GM8: Post hoc analysis | n | n | n | n | n | y | n | n | n | n | n | n | n | n | n | n | n | n | n | n |
| GR1: Missing data | n | n | n | n | n | y | n | n | n | n | n | n | n | n | n | y | n | n | n | n |
| GR2: Participant/patient Characteristics | y | y | y | y | y | y | y | y | y | y | y | y | y | y | y | y | y | y | y | y |
| GR3: Sample size | y | y | y | y | y | y | y | y | y | y | y | y | y | y | y | y | y | y | y | y |
| D1: MP evidence | y | y | y | y | y | y | y | y | y | y | y | y | y | y | y | y | y | y | y | y |
| D2: Practical relevance | y | y | y | y | y | y | y | y | y | y | y | y | y | y | y | y | y | y | y | y |
| D3: Strengths and limitations | y | y | y | y | y | y | y | y | y | y | y | y | y | y | y | y | y | y | y | y |
| D4: Generalizability | y | y | y | y | y | y | y | y | y | y | y | y | y | y | y | y | y | y | y | y |
| D5: Instrument changes | y | y | y | y | y | y | y | n | y | n | y | n | n | n | y | y | n | y | y | n |
| D6: Future Research | y | y | y | n | y | y | y | y | y | y | y | y | y | y | y | y | y | y | y | y |
| C1: Conclusions | n | y | y | y | y | y | y | y | y | y | y | y | y | y | y | y | y | y | y | y |
| O1: Conflict of interest | n | y | n | y | y | y | n | n | n | y | n | n | y | n | n | n | y | n | y | n |

y=yes, n=no, uc=unclear

T1-T3 refers to information provided in the title of the study.

A1-A7 focus of information in the abstract.

I1-I6 are contents of the introduction.

GM1-GM8 focus on method section of the study.

GR1-GR3 focus on general results of the study.

D1-D6 focus on reporting the discussion.

C1 focus on conclusions.

O1 focus on describing conflict of interest.
