# Supplementary material for: Introduction and Systematic Review of the Good Nursing Care Scale
Source: J Clin Nurs. 2024 Oct 11;34(1):5–23. doi: 10.1111/jocn.17486 (PMC11655425; doi:10.1111/jocn.17486)
Supplement: Supplementary file 4 — Appendix S4 [file JOCN-34-5-s003.docx]

Appendix S4. Level of the quality of nursing care in the studies using Good Nursing Care Scale

| **Studies** | **Nursing staff characteristics** | | | **Nursing care activities** | | | **Preconditions for care** | | | **Nursing environment** | | | **Proceeding of the nursing process** | | | **Patients’ empowerment**  **strategies** | | | **Collaboration with family members** | | | **Total** | | |
| --- | --- | --- | --- | --- | --- | --- | --- | --- | --- | --- | --- | --- | --- | --- | --- | --- | --- | --- | --- | --- | --- | --- | --- | --- |
|  | M | Sd | n | M | Sd | n | M | Sd | n | M | Sd | n | M | Sd | n | M | Sd | n | M | Sd | n | M | Sd | n |
| The Good Perioperative Nursing Care Scale (GPNCS) ©Leinonen, 54-item version, scale 1–5 | | | | | | | | | | | | | | | | | | | | | | | | |
| **1a** | 4.82 | 0.32 | 778 | 4.48 | 0.48 | 377 | 4.61 | 0.58 | 675 | 4.78 | 0.38 | 708 | 4.64 | 0.61 | 743 |  |  |  |  |  |  | 4.7 |  |  |
| **1c** | 4.82 | 0.32 | 778 | 4.48 | 0.48 | 377 | 4.61 | 0.58 | 675 | 4.78 | 0.38 | 708 | 4.64 | 0.61 | 743 |  |  |  |  |  |  |  |  |  |
| The Good Perioperative Nursing Care Scale (GPNCS) ©Leinonen, 34-item version, scale 32–160 (sum of the results) | | | | | | | | | | | | | | | | | | | | | | | | |
| **1e** | 23.9 | 2.0 | 215 |  |  |  |  |  |  | 18.8 | 1.8 | 215 |  |  |  |  |  |  |  |  |  | 146.6 | 14.0 | 215 |
| **1f** | 17.77 | 2.39 | 90 |  |  |  |  |  |  | 18.02 | 2.00 | 90 | 8.79 | 1.43 | 90 |  |  |  |  |  |  | 134.61 | 16.29 | 90 |
| Version 1 from 1994, scale 1–6 (reversed to the others) | | | | | | | | | | | | | | | | | | | | | | | | |
| **3** | 3.35 | 0.46 | 178 |  |  |  | 3.15 | 0.59 | 120 | 3.18 | 0.56 | 164 |  |  |  |  |  |  |  |  |  |  |  |  |
|  | 3.48 | 0.47 | 115 |  |  |  | 3.29 | 0.64 | 88 | 3.54 | 0.39 | 106 |  |  |  |  |  |  |  |  |  |  |  |  |
|  | 3.65 | 0.34 | 286 |  |  |  | 3.54 | 0.43 | 262 | 3.61 | 0.38 | 272 |  |  |  |  |  |  |  |  |  |  |  |  |
|  | 3.68 | 0.34 | 255 |  |  |  | 3.54 | 0.39 | 228 | 3.62 | 0.36 | 247 |  |  |  |  |  |  |  |  |  |  |  |  |
| **4** | 2.54 | 0.55 | 100 | 1.97 | 0.78 | 100 | 2.28 | 0.7 | 100 | 2.82 | 0.40 | 100 | 1.34 | 0.88 | 100 |  |  |  | 1.47 | 2.03 | 100 |  |  |  |
| Child Care Quality at Hospital (CCQH) ©Pelander, scale 1–3 in “Nursing staff characteristics” and “Nursing care activities”, scale 1–4 in “Nursing environment” | | | | | | | | | | | | | | | | | | | | | | | | |
| **5a** | 2.66 | 0.24 | 388 | 2.48 | 0.29 | 385 |  |  |  | 3.18 | 0.44 |  |  |  |  |  |  |  |  |  |  |  |  |  |
| **5b** | 2.66 | 0.24 | 388 | 2.48 | 0.29 | 385 |  |  |  | 3.18 | 0.44 |  |  |  |  |  |  |  |  |  |  |  |  |  |
| Child Care Quality at Hospital (CCQH) ©Pelander, scale 1–5 | | | | | | | | | | | | | | | | | | | | | | | | |
| **5d** | 3.79 | 1.07 | 692 | 3.03 | 0.96 | 692 |  |  |  | 2.78 | 0.64 | 692 |  |  |  |  |  |  |  |  |  | 3.96 | 0.93 | 692 |
| Version 1 from 1994, scale 1–4 (reversed to the others) | | | | | | | | | | | | | | | | | | | | | | | | |
| **6a** | 1.15 | 0.25 | 131 | 1.38 | 0.35 | 130 |  |  |  | 1.42 | 0.26 | 129 | 1.37 | 0.34 | 129 |  |  |  |  |  |  |  |  |  |
| **6b** | 1.15 | 0.25 | 131 | 1.38 | 0.35 | 130 |  |  |  | 1.42 | 0.26 | 129 | 1.37 | 0.34 | 129 |  |  |  |  |  |  | 1.33 |  |  |
| Version 2.1 from 2008, scale 1–5 | | | | | | | | | | | | | | | | | | | | | | | | |
| **7** | 4.14 | 0.58 | 383 | 4.12 | 0.56 | 383 | 4.11 | 0.69 | 383 | 4.16 | 0.61 | 383 | 4.17 | 0.62 | 383 |  |  |  | 4.13 | 0.63 | 383 |  |  |  |
| **11b** | 3.28 | 0.90 | 200 | 2.98 | 0.94 | 200 | 2.83 | 1.02 | 200 | 3.26 | 0.84 | 200 | 2.74 | 0.94 | 200 | 2.37 | 1.02 | 200 | 2.47 | 1.22 | 200 | 2.81 | 0.75 | 200 |
| Version 2.0 from 2008, scale 1–6 | | | | | | | | | | | | | | | | | | | | | | | | |
| **8** | 5.44 | 0.75 | 1063 |  |  |  | 5.15 | 0.96 | 1059 | 5.36 | 0.97 | 1056 | 4.45 | 0.99 | 969 |  |  |  | 4.55 | 1.36 | 679 |  |  |  |
| **9** | 5.46 | 0.67 | 226 | 5.18 | 0.87 | 222 | 5.43 | 0.64 | 217 | 5.41 | 0.61 | 223 | 5.09 | 0.81 | 219 | 5.17 | 0.85 | 210 |  |  |  | 5.29 | 0.65 | 226 |
| Version 3 from 2013, scale 1–4 | | | | | | | | | | | | | | | | | | | | | | | | |
| **10a** | 3.83 | 0.33 | 236 | 3.71 | 0.40 | 234 | 3.75 | 0.40 | 230 | 3.85 | 0.32 | 235 | 3.66 | 0.42 | 236 | 3.57 | 0.47 | 232 | 3.11 | 0.84 | 213 | 3.45 | 0.32 | 238 |
|  | 3.79 | 0.40 | 180 | 3.65 | 0.48 | 179 | 3.65 | 0.50 | 175 | 3.81 | 0.30 | 181 | 3.67 | 0.37 | 178 | 3.50 | 0.54 | 174 |  |  |  | 3.43 | 0.34 | 182 |
| **10b** | 3.82 | 0.35 | 470 | 3.70 | 0.43 | 467 | 3.72 | 0.44 | 453 | 3.84 | 0.30 | 466 | 3.70 | 0.40 | 459 | 3.55 | 0.50 | 447 | 3.00 | 0.93 | 316 | 3.47 | 0.34 | 476 |
| **12** | 3.70 | 0.50 | 85 | 3.55 | 0.53 | 85 | 3.57 | 0.50 | 85 | 3.79 | 0.30 | 85 | 3.45 | 0.52 | 85 | 3.43 | 0.59 | 85 | 2.72 | 0.94 | 85 | 3.49 | 0.42 | 85 |
|  | 3.81 | 0.33 | 238 | 3.70 | 0.41 | 238 | 3.69 | 0.39 | 238 | 3.86 | 0.26 | 238 | 3.65 | 0.43 | 238 | 3.57 | 0.48 | 238 | 2.92 | 0.92 | 238 | 3.63 | 0.35 | 238 |

M = mean; Sd = standard deviation
